# Biofilm Degradation by Seashell-Derived Calcium Hydroxide and Hydrogen Peroxide

**DOI:** 10.3390/nano12203681

**Published:** 2022-10-20

**Authors:** Yuuki Hata, Yuta Bouda, Sumiyo Hiruma, Hiromi Miyazaki, Shingo Nakamura

**Affiliations:** Division of Biomedical Engineering, National Defense Medical College Research Institute, 3-2 Namiki, Tokorozawa-shi 359-8513, Japan

**Keywords:** biofilm, extracellular polymeric substance, degradation, calcium hydroxide, seashell, hydrogen peroxide

## Abstract

Microbial cells and self-produced extracellular polymeric substances assembled to form biofilms that are difficult to remove from surfaces, causing problems in various fields. Seashell-derived calcium hydroxide, a sustainable inorganic material, has shown high bactericidal activity even for biofilms due to its alkalinity. However, its biofilm removal efficacy is relatively low. Herein, we report a biofilm degradation strategy that includes two environmentally friendly reagents: seashell-derived calcium hydroxide and hydrogen peroxide. A biofilm model of *Escherichia coli* was prepared in vitro, treated with calcium hydroxide–hydrogen peroxide solutions, and semi-quantified by the crystal violet stain method. The treatment significantly improved biofilm removal efficacy compared with treatments by calcium hydroxide alone and hydrogen peroxide alone. The mechanism was elucidated from calcium hydroxide–hydrogen peroxide solutions, which suggested that perhydroxyl anion and hydroxyl radical generated from hydrogen peroxide, as well as the alkalinity of calcium hydroxide, enhanced biofilm degradation. This study showed that concurrent use of other reagents, such as hydrogen peroxide, is a promising strategy for improving the biofilm degradation activity of seashell-derived calcium hydroxide and will contribute to developing efficient biofilm removal methods.

## 1. Introduction

Biofilms are assemblies of microbial cells and self-produced extracellular polymeric substances, including polysaccharides and other biopolymers [1,2,3,4,5]. This lifestyle of bacteria is found on surfaces of living tissues, indwelling medical devices, industrial or potable water system piping, and natural aquatic systems. The biofilm state renders microbial cells highly resistant to harsh environmental factors, such as extreme temperature, extreme pH, high salinity, ultraviolet radiation, lack of nutrients, and antibiotics. Moreover, biofilms are sticky and viscoelastic due to the supramolecular structure of extracellular polymeric substances and therefore difficult to remove from surfaces [6]. Consequently, biofilms cause various problems in medical fields and food and other industries [7,8,9,10]. Harsh chemicals, such as chlorine, sodium hypochlorite, and other oxidizing agents, are typically required to kill and remove biofilms [9,11]. These reagents may damage tissues and materials, on which biofilms are attached, and produce harmful byproducts (e.g., trihalomethane) [12]. The development of effective and safe methods to eradicate biofilms remains challenging.

Recent studies have used calcium hydroxide (Ca(OH)_2_) and calcium oxide (CaO) prepared from seashells to develop effective and safe disinfectants [13,14]. Seashells are mainly composed of calcium carbonate and can be converted into CaO via heat treatments [13]. Subsequent hydration of CaO produces Ca(OH)_2_. These seashell-derived Ca(OH)_2_ and CaO have shown excellent microbicidal and virucidal activities due to their alkalinity [13,14]. Thus, their aqueous solutions, dispersions, and suspensions have been investigated as sustainable and halogen-free disinfectants [15,16,17,18,19,20,21]. Indeed, seashell-derived Ca(OH)_2_ and CaO can kill not only planktonic bacterial cells, but also biofilms [22,23,24], and thus they are promising for developing sustainable and halogen-free biofilm treatment methods. Nevertheless, the biofilm removal efficacy of seashell-derived Ca(OH)_2_ and CaO is relatively low despite their excellent bactericidal activities. Killed biofilms can still discharge endotoxins and facilitate the recolonization of new and viable biofilm [25,26,27,28]. Hence, methods to enhance the biofilm removal efficacy of seashell-derived Ca(OH)_2_ and CaO are highly sought.

Herein, we found that Ca(OH)_2_ and hydrogen peroxide (H_2_O_2_) synergize to effectively remove biofilm. H_2_O_2_ is an oxidizing agent that generates only water and oxygen as byproducts and is widely used for disinfection and bleaching [29,30,31]. Notably, alkaline conditions enhance the activity of H_2_O_2_: H_2_O_2_ is converted to perhydroxyl anion (HOO^−^) and subsequently to hydroxyl radical (HO⋅) and other reactive species [29,30,32]. Thus, we hypothesized that the addition of H_2_O_2_ to Ca(OH)_2_ alkaline solutions would enhance biofilm removal efficacy. A model biofilm of *Escherichia coli* (*E. coli*) was prepared on polypropylene surfaces and treated with Ca(OH)_2_–H_2_O_2_ aqueous solutions. This treatment significantly decreased the amount of biofilms on the surface despite a slight decrease in the alkalinity of Ca(OH)_2_ by weakly acidic H_2_O_2_.

## 2. Materials and Methods

### 2.1. Materials

Ethanol, Ca(OH)_2_, and ~30 wt% H_2_O_2_ were purchased from FUJIFILM Wako Pure Chemical Corporation (Osaka, Japan). Brain heart infusion broth, Gram-Hucker’s stain solution I (20% ethanol, 2% crystal violet, and 0.8% ammonium oxalate), 5,5-dimethyl-1-pyrroline *N*-oxide, and saline were purchased from Nissui Pharmaceutical (Tokyo, Japan), Muto Pure Chemicals (Tokyo, Japan), Labotec (Tokyo, Japan), and Otsuka Pharmaceutical Factory (Tokushima, Japan), respectively. *E. coli* (ATCC 51813) was obtained from Microbiologics (St Cloud, MN, USA). Scallop shell-derived Ca(OH)_2_ was kindly donated by Hokkaido Lime (Hokkaido, Japan). Ultrapure water with a resistivity greater than 18.2 MΩ cm at 25 °C was supplied by an RFU464TA instrument (Advantec, Tokyo, Japan).

### 2.2. Preparation of Biofilms

Two hundred microliters of *E. coli* suspensions (ATCC 51813, ~1 × 10^7^ colony-forming units mL^−1^) in 1/5 brain heart infusion broth (7 g L^−1^) as the medium were added to each well of a 96-well polystyrene microplate (Thermo Fisher Scientific, Waltham, MA, USA). The microplates were then covered with 96-pin polypropylene lids (BMR-PP, BM Equipment, Tokyo, Japan) and incubated at 37 °C for 1 d to allow biofilms to form on the pins. The 96-pin lids containing biofilms were transferred to a 96-well plate containing fresh 1/5 brain heart infusion broth and incubated at 37 °C for 2 d to further grow biofilms. The medium was refreshed after 1 d.

### 2.3. Degradation and Semi-Quantification of Biofilms

After washing with saline, the 96-pin lids containing biofilms were transferred to 96-well microplates containing 250 µL of Ca(OH)_2_–H_2_O_2_ aqueous solutions in each well. The biofilms in Ca(OH)_2_–H_2_O_2_ solutions were incubated at room temperature for 10 min unless otherwise stated. After washing with saline, the 96-pin lids were transferred to 96-well microplates containing 250 µL of Gram-Hucker’s stain solution I in each well and incubated for 20 min. Stained biofilms were washed three times with saline and dried for more than 30 min. The dye crystal violet was extracted with ethanol (250 µL for each pin), followed by 10-fold dilution, with subsequent absorbance measurements performed at 595 nm using a Multiskan FC microplate reader (Thermo Fisher Scientific, Massachusetts, USA). The experiment was repeated three times independently. Statistical analysis was performed by parametric one-way analysis of variance (ANOVA) with Dunnett’s multiple comparison using GraphPad Prism9 software (GraphPad Software, San Diego, CA, USA).

### 2.4. Microscopic Observations of Biofilms

For scanning electron microscopy (SEM), the biofilms on pins were fixed with 0.1 M phosphate buffer solutions (pH 7.4) containing 2% paraformaldehyde and 2.5% glutaraldehyde, and subsequently with 0.1 M phosphate buffer solutions (pH 7.4) containing 1% osmium tetroxide. The samples were solvent-exchanged with 50, 70, and 95% ethanol, ethanol, ethanol dehydrated with a molecular sieve, and then isoamyl acetate. After critical point drying using an HCP-2 instrument (Hitachi, Tokyo, Japan), the samples were mounted on substrates using silver paste and coated with osmium. The surfaces were observed with a JSM-6340F instrument (JEOL, Tokyo, Japan) at an accelerating voltage of 5 kV.

For fluorescence microscopy, the biofilms on pins before and after the treatment with Ca(OH)_2_–H_2_O_2_ solutions were stained using LIVE/DEAD *Bac*Light Bacterial Viability Kit (Thermo Fisher Scientific, Waltham, MA, USA). Specifically, the pins with biofilms were immersed in aqueous solutions of 4.8 µM SYTO 9 and 24 µM propidium iodide for 15 min in the dark. The samples were observed by a BZ-X800 instrument equipped with a Plan Apochromat 2x objective (Keyence, Osaka, Japan). Observations were conducted using an OP-87763 filter (excitation: 470 nm, absorption: 525 nm), an OP-87764 filter (excitation: 545 nm, absorption: 605 nm) for SYTO 9, and propidium iodide, respectively, at an exposure time of 1/6 s.

### 2.5. Characterization of Ca(OH)_2_–H_2_O_2_ Solutions

For pH measurements, 250 µL of Ca(OH)_2_–H_2_O_2_ aqueous solutions was prepared immediately before measurements. A pH meter F-74 equipped with a 9618S electrode (Horiba, Kyoto, Japan) was operated at room temperature.

For electron spin resonance (ESR) spectroscopy, Ca(OH)_2_ aqueous solutions and H_2_O_2_ aqueous solutions were mixed to prepare 0.1% Ca(OH)_2_–0.1% H_2_O_2_ solutions before incubation at room temperature for 1, 10, and 60 min. Fifty microliters of the mixtures were mixed with 10 µL of a radical trapping agent, 5,5-dimethyl-1-pyrroline *N*-oxide. An EMX-nano spectrometer (Bruker, Billerica, MA, USA) was operated at 9.65 GHz microwave frequency, 10 mV microwave power, and 5 scans.

## 3. Results and Discussion

### 3.1. Biofilm Preparation

**Figure 1:**
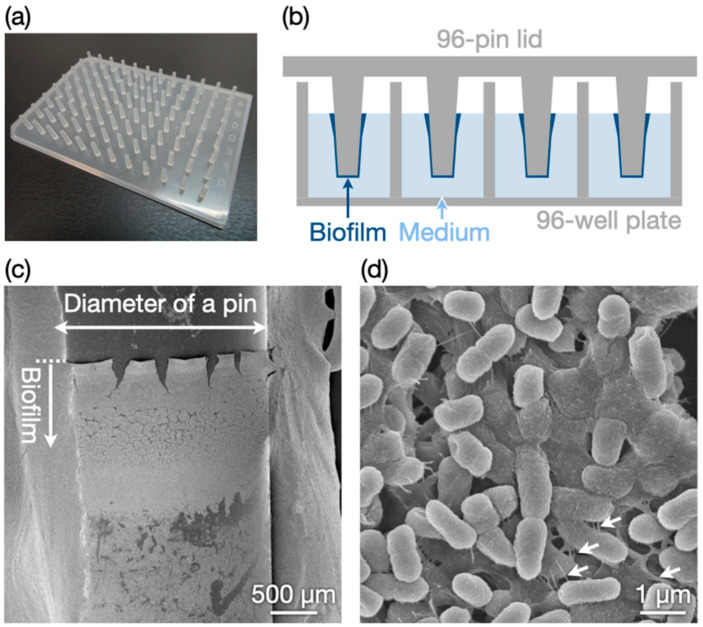
Preparation of an in vitro biofilm model. (**a**) Photograph of the 96-pin lid. (**b**) Schematic illustration of biofilm preparation. SEM images of prepared biofilms at (**c**) low and (**d**) high magnifications. The dotted line in (**c**) indicates the upper edge of the biofilms. The arrows in (**d**) indicate nanofibrous materials.

We adapted the in vitro biofilm preparation method using 96-pin plastic lids [33,34]. A 96-well microplate containing *E. coli* in a diluted liquid medium was covered with a 96-pin lid (Figure 1a) and incubated for 3 d. Biofilms formed on the pins in the liquid medium, as illustrated in Figure 1b. Notably, the biofilms were relatively thick at the upper edge near the air (Figure 1c). Figure 1d is an SEM image at a high magnification that revealed densely packed bacterial cells and nanofibrous materials around the cells. The nanofibrous materials appeared to be extracellular polymeric substances and/or flagella. The results confirmed the successful preparation of an in vitro biofilm model.

### 3.2. Biofilm Degradation by Ca(OH)_2_–H_2_O_2_

**Figure 2:**
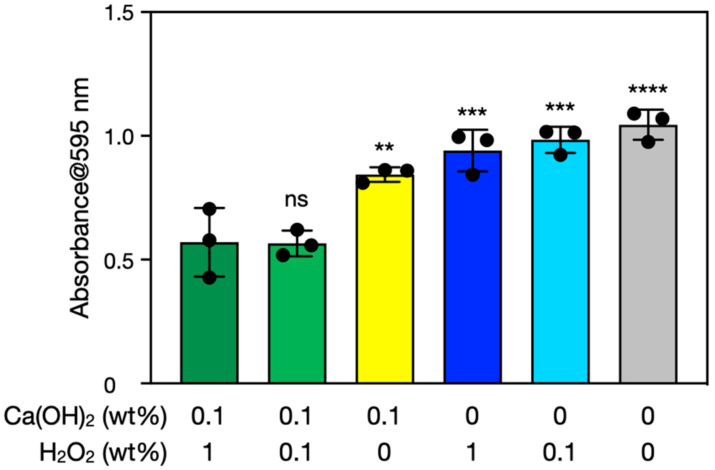
Semi-quantification of biofilms before and after the treatments with Ca(OH)_2_ and/or H_2_O_2_ for 10 min. The *y*-axis is the 595-nm absorbance of crystal violet extracted from the stained biofilms. The error bars represent the standard deviation of three individual trials. Statistical analysis was performed by parametric one-way ANOVA with Dunnett’s multiple comparison; ns, **, ***, and **** denote not significant, *p* < 0.01, *p* < 0.001, and *p* < 0.0001, respectively.

The prepared biofilms were immersed in Ca(OH)_2_–H_2_O_2_ solutions and incubated at room temperature for 10 min. Reagent-grade Ca(OH)_2_, rather than seashell-derived Ca(OH)_2_, was initially used for fundamental investigations before experiments using seashell-derived Ca(OH)_2_ (see Section 3.4). The amounts of biofilms were semi-quantified with the crystal violet stain method [35,36]. This method enabled us to semi-quantify biomolecules of bacterial cells and extracellular polymeric substances by measuring the absorbance of crystal violet extracted from the stained biofilms. Figure 2 shows results of biofilm semi-quantification before and after the treatments with Ca(OH)_2_ and/or H_2_O_2_ for 10 min. Solutions containing 0.1% Ca(OH)_2_ and 0.1% to 1% H_2_O_2_ significantly decreased the amount of biofilm on the surface. These decreases correspond to the removal of biofilms rather than bacterial death. Ca(OH)_2_–H_2_O_2_ removed significantly more biofilm than Ca(OH)_2_ alone or H_2_O_2_ alone. Thus, the concurrent use of H_2_O_2_ improves the biofilm removal efficacy of Ca(OH)_2_.

**Figure 3:**
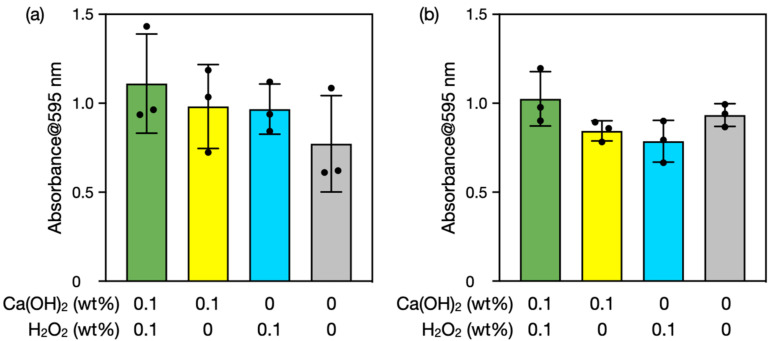
Semi-quantification of biofilms before and after the treatments with Ca(OH)_2_ and/or H_2_O_2_ for (**a**) 3 and (**b**) 30 min. The *y*-axis is the 595-nm absorbance of crystal violet extracted from the stained biofilms. The error bars represent the standard deviation of three individual trials.

Ca(OH)_2_–H_2_O_2_ treatment time was varied from 10 min to 3 and 30 min (Figure 3). One-way ANOVA indicated nonsignificant differences between the groups (*p* > 0.05). The amount of biofilm hardly decreased after treatments for 3 and 30 min even with the use of both Ca(OH)_2_ and H_2_O_2_. The shorter treatment time proved insufficient to degrade biofilms. Meanwhile, the longer treatment time may have enabled readsorption of extracellular polymeric substances due to decreases in pH and reactive species concentrations (see Section 3.3). Thus, 10 min of treatment was considered optimal for biofilm degradation under the conditions investigated.

**Figure 4:**
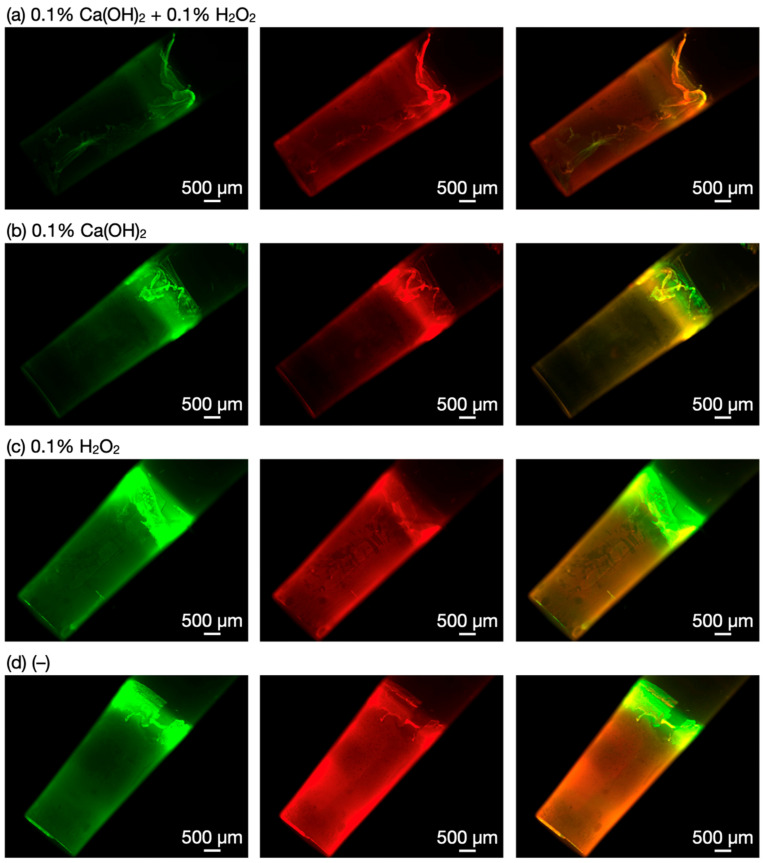
Fluorescence microscopy images of biofilms after the treatments with (**a**) 0.1% Ca(OH)_2_–0.1% H_2_O_2_, (**b**) 0.1% Ca(OH)_2_, and (**c**) 0.1% H_2_O_2_ and (**d**) nontreated biofilms. Each image visualizes a polypropylene pin with biofilms. The green fluorescence (left) and the red fluorescence (middle) correspond to SYTO 9 and propidium iodide, respectively. Merged images of the two channels are shown in the right column.

The biofilms treated with Ca(OH)_2_ and/or H_2_O_2_ were stained with SYTO 9 and propidium iodide for fluorescence microscopy. SYTO 9, a green fluorescence dye, permeates both intact and damaged membranes of the bacterial cells, and binds to nucleic acids. Meanwhile, propidium iodide, a red fluorescence dye, permeates only damaged membranes, and binds with a higher affinity to nucleic acids than SYTO 9. It is noted that extracellular polymeric substances should be non-specifically stained by these fluorescence dyes to a certain extent, causing background fluorescence. Fluorescence microscopy images of the treated biofilms are shown in Figure 4. The biofilms treated with Ca(OH)_2_–H_2_O_2_ exhibited less fluorescence of both SYTO 9 and propidium iodide than the other biofilm samples, indicating that the Ca(OH)_2_–H_2_O_2_ treatment killed bacteria and removed dead cells, which was also suggested by the crystal violet stain method.

### 3.3. Investigations into Ca(OH)_2_–H_2_O_2_ Solutions

**Figure 5:**
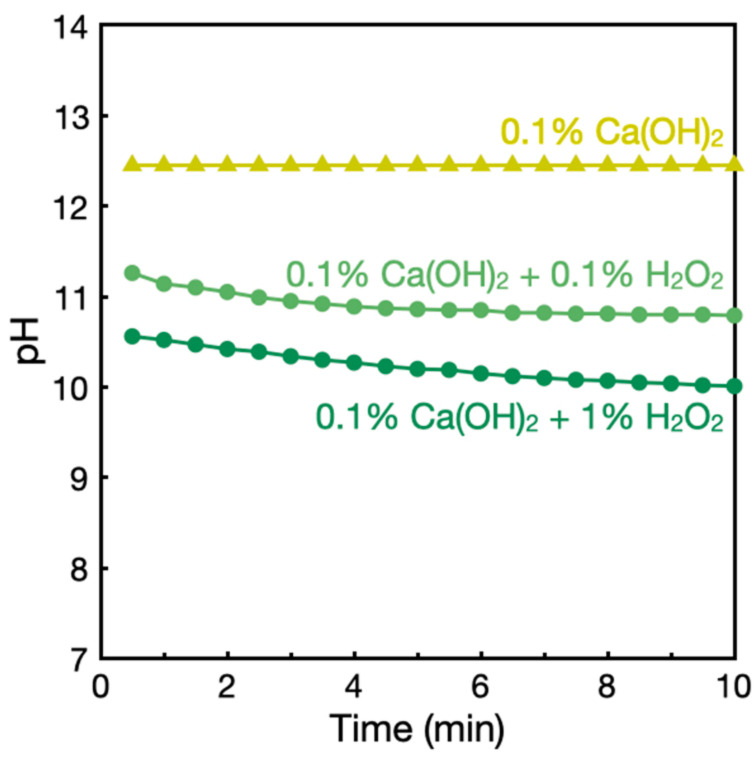
pH time courses of the solutions of Ca(OH)_2_ and H_2_O_2_.

We characterized Ca(OH)_2_–H_2_O_2_ solutions to better understand biofilm degradation. Figure 5 presents the time courses of pH of the solutions. The addition of H_2_O_2_ decreased the alkalinity of Ca(OH)_2_ solutions probably due to the reaction of weakly acidic H_2_O_2_ (p*K*_a_ = 11.6) with HO^−^, producing HOO^−^. HOO^−^ is a reactive species responsible for the bleaching action of H_2_O_2_ under alkaline conditions [29]. The pH further gradually decreased (Figure 5), which can be attributed to the decomposition of HOO^−^ and subsequent reproduction of HOO^−^ by consuming HO^−^. Considering that a decrease in pH should reduce the bactericidal and biofilm removal activities of Ca(OH)_2_ derived mainly from its alkalinity, the improved biofilm removal activities of Ca(OH)_2_–H_2_O_2_ solutions seemed to originate from not only HO^−^, but also reactive species generated from H_2_O_2_, such as HOO^−^.

**Figure 6:**
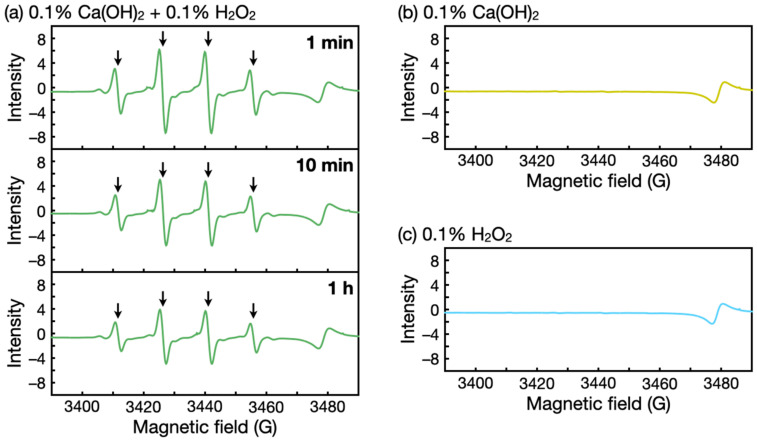
ESR spectra of (**a**) Ca(OH)_2_–H_2_O_2_ solutions, (**b**) Ca(OH)_2_ solutions, and (**c**) H_2_O_2_ solutions. The arrows in (**a**) indicate characteristic signals of HO⋅.

Other possible active species other than HOO^−^ include HO⋅, a highly reactive species that forms via a reaction between HOO^−^ and H_2_O_2_ [29,32]. Thus, we conducted ESR spectroscopy measurements using 5,5-dimethyl-1-pyrroline *N*-oxide as the radical trapping agent. ESR spectra of Ca(OH)_2_–H_2_O_2_ solutions showed four characteristic signals of HO⋅ (Figure 6a). These peaks were absent in spectra of Ca(OH)_2_ alone and H_2_O_2_ alone (Figure 6b,c). These results indicate that H_2_O_2_ with alkaline Ca(OH)_2_ generated highly reactive HO⋅, which contributed to the biofilm degradation. The HO⋅ signal intensity also gradually decreased (Figure 6a). This decrease in HO⋅ implies the decomposition of H_2_O_2_ over time and may explain the decreased biofilm removal efficacy at 30 min of the treatment (Figure 3b).

H_2_O_2_ decomposes to produce not only reactive species but also oxygen bubbles that may help remove biofilms via mechanical disruption [37]. Collectively, Ca(OH)_2_–H_2_O_2_ solutions, albeit weaker alkaline solutions than Ca(OH)_2_ solutions, better remove biofilm due to the synergy of HO^−^, HOO^−^, HO⋅, and possibly oxygen bubbles. Given the suggested mechanism based on chemically active species, the biofilm removal method using Ca(OH)_2_ and H_2_O_2_ will be effective to various bacteria strains and even species.

### 3.4. Biofilm Degradation Using Seashell-Derived Ca(OH)_2_

**Figure 7:**
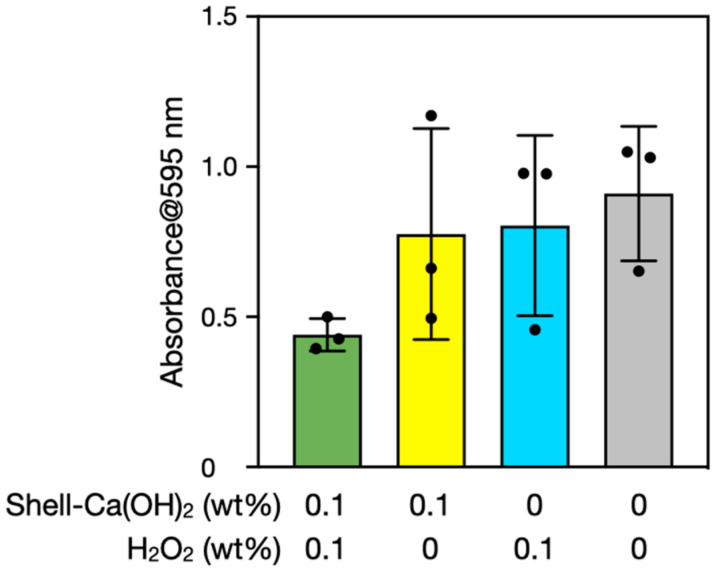
Semi-quantification of biofilms before and after the treatments with scallop shell-derived Ca(OH)_2_ and/or H_2_O_2_ for 10 min. The *y*-axis is the 595-nm absorbance of crystal violet extracted from the stained biofilms. The error bars represent the standard deviation of three individual trials.

Finally, we performed experiments using scallop shell-derived Ca(OH)_2_ because naturally derived materials may contain impurities that influence H_2_O_2_ reactivities. Biofilms were therefore treated with scallop shell-derived Ca(OH)_2_ rather than reagent-grade Ca(OH)_2_. Figure 7 shows the results of biofilm semi-quantification by the crystal violet stain method before and after the treatments. One-way ANOVA indicated nonsignificant differences between the groups (*p* > 0.05). Nevertheless, average values showed a trend similar to those of the results using reagent-grade Ca(OH)_2_ (Figure 2), suggesting that scallop shell-derived Ca(OH)_2_ also has the potential to remove biofilms with the concurrent use of H_2_O_2_. Available natural sources of Ca(OH)_2_ include seashells, eggshells, and limestone.

## 4. Conclusions

We used Ca(OH)_2_ and H_2_O_2_ to degrade biofilms. This mixture removed a significantly greater amount of biofilm from the surface than Ca(OH)_2_ alone and H_2_O_2_ alone. Further, the 10 min treatment was considered optimal for biofilm degradation under the conditions investigated, as shorter and longer treatment time resulted in decreased efficacy. The higher biofilm degradation activity of Ca(OH)_2_–H_2_O_2_ solutions was attributed to the synergy of HO^−^ from Ca(OH)_2_ with reactive species from H_2_O_2_, such as HOO^−^ and HO⋅. The potential byproducts are environmentally friendly: CaCO_3_ and water from Ca(OH)_2_ and oxygen and water from H_2_O_2_. Our findings suggest that the concurrent use of other reagents, such as H_2_O_2_, is a promising strategy for improving the biofilm degradation activity of seashell-derived Ca(OH)_2_, and will inspire the development of more efficient biofilm removal methods.

## Data Availability

Not applicable.
